# Correction: Open-source food: Nutrition, toxicology, and availability of wild edible greens in the East Bay

**DOI:** 10.1371/journal.pone.0239794

**Published:** 2020-09-22

**Authors:** Philip B. Stark, Daphne Miller, Thomas J. Carlson, Kristen Rasmussen de Vasquez

There is an error in Table 8. The values 10.94 oxalic acid-soluble (mg/g) and 15.42 oxalic acid-total (mg/g) should appear under the heading oxalis *Oxalis pes-caprae* instead of the heading nasturtium *Tropaelolum majus*. Please see the correct Table 8 here.

**Table 8:** Nutritional tests of wet plant tissue (performed by SCS Global Services in Emeryville, CA) collected by Berkeley Open Source Food in West Oakland, CA, and USDA National Nutrient Database values for raw kale.

	chickweed *Stellaria* *media*	dandelion *Taraxacum* *officinale*	dock *Rumex* *crispus*	mallow *Malva* *sylvestris*	nasturtium *Tropaeolum* *majus*	oxalis O*xalis* *pes-caprae*	kale *Brassica* *oleraceae*
cal (Kcal)	29.09	34.86	33.37	52.14	46.91	27.52	35.0
fat cal (Kcal)	2.40	3.47	2.47	3.58	6.39	2.52	13.41
fat (g)	0.27	0.39	0.27	0.40	0.71	0.28	1.49
saturated fat (g)	0.01	0.01	0.02	0.01	0.04	0.01	0.18
TFA (g)	0	0	0	0	0	0	0
cholesterol (mg)	0	0	0	0	0	0	0
carbohydrates (g)	5.19	5.55	4.79	7.81	6.90	5.27	4.42
dietary fiber (g)	3.64	5.26	3.39	7.20	3.10	2.99	4.10
total sugars (g)	0	0	0	0	0.37	0	0.99
protein (g)	1.43	2.27	2.63	4.10	3.23	0.98	2.92
Vitamin A (IU)	2282	6577	5396	4637	8182	2369	4812
Vitamin C (mg)	10.66	4.49	36.19	8.65	1.49	9.40	93.40
Na (mg)	45.17	52.34	101.04	42.87	39.97	28.85	53.0
Ca (mg)	65.96	95.90	68.47	273.39	148.46	48.69	254.0
Fe (mg)	1.54	2.73	1.31	3.35	1.18	1.87	1.60
K (mg)	439.82	440.08	310.24	357.09	297.97	128.29	348.0
total phenolics (mg/g)	0.77	0.49	2.77	1.29	2.82	1.68	NA
oxalic acid-soluble (mg/g)			0.18			10.94	
oxalic acid-total (mg/g)			0.39			15.42	

Results are per 100g of wet tissue except total phenolics and oxalic acid, which are concentrations (mg/g) See Table 6 for sample locations.

## References

[1] Stark PB, Miller D, Carlson TJ, de Vasquez KR (2019) Open-source food: Nutrition, toxicology, and availability of wild edible greens in the East Bay. PLoS ONE 14(1): e0202450 10.1371/journal.pone.0202450 30653545 PMC6336281

